# Natural Halloysites-Based Janus Platelet Surfactants for the Formation of Pickering Emulsion and Enhanced Oil Recovery

**DOI:** 10.1038/s41598-018-36352-w

**Published:** 2019-01-17

**Authors:** Lecheng Zhang, Qun Lei, Jianhui Luo, Minxiang Zeng, Ling Wang, Dali Huang, Xuezhen Wang, Sam Mannan, Baoliang Peng, Zhengdong Cheng

**Affiliations:** 1 Artie McFerrin Department of Chemical Engineering, Texas A&M University, College Station, TX 77843-3122 USA; 2 Mary Kay O’Connor Process Safety Center, Artie McFerrin Department of Chemical Engineering, Texas A&M University, College Station, TX 77843-3122 USA; 3 Department of Materials Science & Engineering, Texas A&M University, College Station, TX 77843-3003 USA; 4 Research Institute of Petroleum Exploration & Development (RIPED), PetroChina, Beijing, 100083 China; 5 Key Laboratory of Nano Chemistry (KLNC), CNPC, Beijing, 100083 China

## Abstract

Janus colloidal surfactants with opposing wettabilities are receiving attention for their practical application in industry. Combining the advantages of molecular surfactants and particle-stabilized Pickering emulsions, Janus colloidal surfactants generate remarkably stable emulsions. Here we report a straightforward and cost-efficient strategy to develop Janus nanoplate surfactants (JNPS) from an aluminosilicate nanoclay, halloysite, by stepwise surface modification, including an innovative selective surface modification step. Such colloidal surfactants are found to be able to stabilize Pickering emulsions of different oil/water systems. The microstructural characterization of solidified polystyrene emulsions indicates that the emulsion interface is evenly covered by JNPS. The phase behaviors of water/oil emulsion generated by these novel platelet surfactants were also investigated. Furthermore, we demonstrate the application of JNPS for enhanced oil recovery with a microfluidic flooding test, showing a dramatic increase of oil recovery ratio. This research provides important insights for the design and synthesis of two-dimensional Janus colloidal surfactants, which could be utilized in biomedical, food and mining industries, especially for circumstances where high salinity and high temperature are involved.

## Introduction

Despite the development of renewable energy sources, fossil fuel will remain as the dominant energy resource in the global energy supply for decades. According to U.S. Energy Information Administration’s (EIA) projection, fossil fuels will supply 75 quadrillion (10^15^) BTU of energy by 2050 to meet the world energy demand^1^. On the production side, production from aging wells will be reduced from current levels. Unless certain stimulation measures are taken, production on an aged well will be less economically viable for oil recovery. Enhanced oil recovery (EOR) is a common well stimulation method to increase productivity of oil wells. Conventional EOR is carried out with molecular surfactants; however, harsh formation conditions, such as high temperature or high salinity, can decrease the efficiency of molecular surfactants or polymer systems. Particle-stabilized emulsion, also known as Pickering emulsion, might provide a solution^2^.

The structures of Pickering emulsions and foams are stabilized with solid particles instead of molecular surfactants. Compared to emulsions stabilized by conventional molecular surfactants, Pickering emulsions are more stable due to their resistance to coalescence and Oswald ripening^3–5^, which makes them perfect candidates for industrial application in foods^6,7^, mining^8,9^, pharmaceuticals^10^ and cosmetics^11^. In related research, a variety of nanoparticles with different morphologies have been used to stabilize Pickering emulsions, such as zero-dimensional nanoparticles^12^, one-dimensional nanowires^13^, and one-dimensional nanotubes^14^. Among different particle morphologies, the idea of applying two-dimensional disks or platelets to stabilize emulsion attracts more attention^15–17^. There are several merits for doing so: firstly, the two-dimensional sheets can provide greater surface coverage than other configurations, which can prevent the emulsified phase from diffusing to the continuous phase. Also, the large surface coverage area significantly increases the magnitude of energy required to remove platelets from the interface. Lastly, the two-dimensional configuration has higher efficiency in terms of material utilization compared to spherical or cylindrical geometries^18^. These merits contribute to the enhanced emulsion stability.

Emulsion stability varies directly with interfacial energy, a characteristic which has been well analyzed in other peer works^19,20^. Interfacial energy, in turn, is related not only to particle geometry, but is also controlled by surface wetting property. To minimize interfacial tension, asymmetric surface modification may be applied to satisfy different wetting preferences on corresponding sides of the platelet. Such amphiphilic structure of a nanoplate is also known as a Janus platelet, which has been previously reported by our group^21,22^. Although the amphiphilic platelets have the potential to be used to stabilize emulsion, the current synthesis methods for such platelets is not reliable. Mask-and-modify is a very common strategy for Janus platelet synthesis^23^. Masking can be achieved on either emulsion interfaces^24^ or solid substrates^25^, but regardless of procedure, reaction can be carried out only at the interface, rendering these methods tedious and not suitable for practical use. Modify-then-exfoliate is more efficient than the mask-and-modify method as it allows bulk reactions rather that limiting reactions to a two-dimensional interface. Although the synthesis of Janus platelets by the modify-then-exfoliate method is more efficient, the yield of Janus platelets is limited, and less desired Gemini platelets will be generated^21^. Careful choice of inorganic template will avoid the formation of Gemini platelets^26^. The yield of final product, however, is still limited by the exfoliation and cleaving process. Some researchers^27^ realized the simplicity of employing asymmetrical substrates, such as kaolinite, for Janus particle synthesis. Covalent grafting on both sides was not achieved, however, which may limit the salinity tolerance of the final surfactant product.

Halloysite, a low-cost natural clay nanoscroll, can be extended into nanoplates with specific phosphonic acid modifications^28,29^. Additionally, halloysite nanoplates possess an asymmetrical crystal structure with the alumina on one side and the silica on the other side. This intrinsic asymmetry makes selective surface modification possible and facilitates the fabrication of Janus structure. Phosphonic acids, a type of common surface modifier^30,31^, can specifically react with the alumina side of the halloysite clay, but not the silica side^32–34^. This selective surface modification can change the wetting property on the alumina side and further mask the alumina side of the nanoplate to protect it from later chemical modification, simplifying the modification of silica side.

Here, we present a two-step versatile method to synthesize a new kind of surface-active nanoplate to stabilize emulsions. The nanoplates are acquired by grafting phenyl phosphonic acid on the alumina side of halloysite as the nanoscroll extends after its alumina side reacting with phenyl phosphonic acid. Yet the phosphonic acid modification does not merely flatten the halloysite nanoscrolls, it also serves as the hydrophobic surface modification and a masking layer to protect the alumina from later reactions. A surface-initiated ATRP is furtherly carried out on the silica side to render the surface hydrophilic. The product is characterized with different methods to confirm the Janus modifications. The interface of Pickering emulsion stabilized by such nanoplate surfactant is studied in detail. Furthermore, an application demonstration is conducted with microfluidic chip to explore the possibilities to apply Janus platelet surfactant for enhanced oil recovery.

## Result and Discussion

### Characterizations of Janus Nanoplates

**Figure 1:**
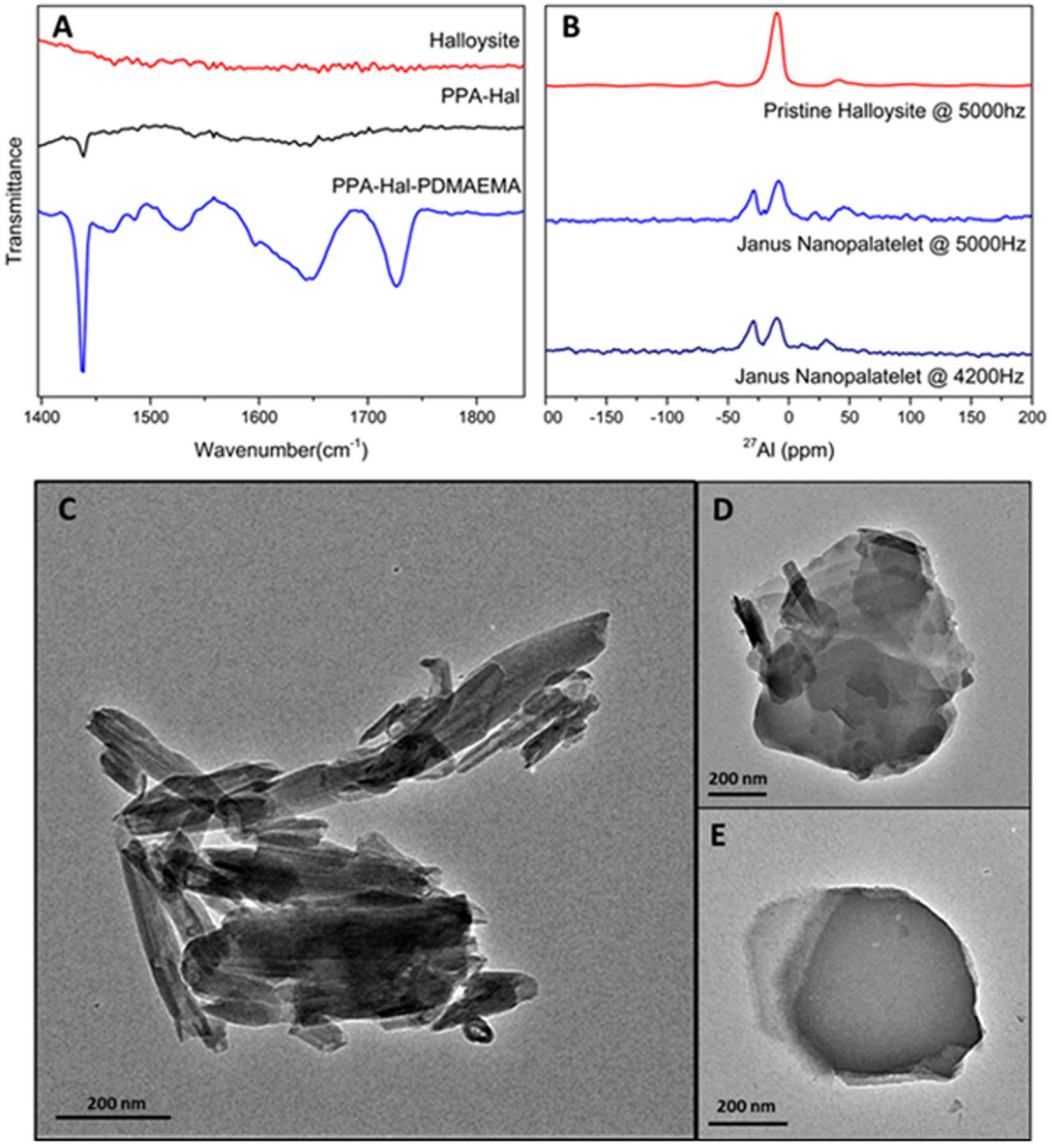
Surface modification characterizations and morphology. (**A**) FT-IR spectra of pristine halloysite (red), PPA-modified halloysite (PPA-halloysite, black), and Janus nanoplate, the asymmetrically modified halloysite (PPA-halloysite-PDMAEMA, blue). After alumina side modification, benzyl characteristic peak (1438 cm^−1^, ring deformation) is observed on the PPA-halloysite spectrum after alumina-side PPA modification. PDMAEMA characteristic peak (1728 cm^−1^, carbonyl stretching) is observed after silica-side ATRP modification; (**B**) ^27^Al MAS ssNMR spectra of pristine halloysite (blue), Janus nanoplate (PPA-halloysite-PDMAEMA, red) @ 5 kHz. Janus nanoplate (PPA-halloysite-PDMAEMA, red) @ 4.2 kHz; transmission electron microscopic images: (**C**) raw halloysite; (**D**) PPA unfolded halloysite nanoplate; (**E**) PPA-halloysite-poly(DMAEMA) nanoplate surfactant.

To confirm the success of surface modification, FT-IR and ssNMR are used to characterize different samples. The ATR FT-IR spectra (Fig. 1A) are obtained with a Thermo Nicolet 380 FT-IR spectrometer. Comparing PPA (phenyl phosphonic acid) -halloysite spectrum to halloysite spectrum, a strong peak appears at 1438 cm^−1^, which is attributed to the stretches of benzene ring. The benzene peak indicates the successful grafting of PPA onto the halloysite sample. Spectrum of PPA-halloysite-PDMAEMA (Poly Dimethylamino ethyl methacrylate) is also collected with similar procedure. Comparing it with the PPA-halloysite and halloysite spectra, the new peak of PPA-halloysite-PDMAEMA at 1728 cm^−1^ is produced by the C = O stretching of DMAEMA polymer. These IR spectra proves the success of the surface modifications on the clay platelets. To further confirm asymmetric modifications on platelets and chemical environmental changes of Al and Si elements after modifications, ssNMR spectra of pristine and modified clay are obtained with a Bruker Avance-400 spectrometer (400 MHz for 1 H nuclei) with a standard 7-mm MAS probe at different spinning rates, as shown in Fig. 1B. The −10 ppm peak in the pristine halloysite corresponds to the chemical environment of Al on the alumina side before modification. After PPA surface modification, another peak at 28 ppm appears, which is attributed to the PPA-modified Al atoms. A couple of satellite peaks are also observed at the 21 ppm and 46 ppm positions. To confirm those satellite peaks, the frequency is changed to 4.2 kHz, we observed shifts of peak positions to 11 ppm and 31 ppm, indicating the presence of satellite speaks. The 28 ppm peak attributed to PPA remains the same, suggesting that the PPA has selectively reacted with the alumina side. However, no chemical shift was observed in the ^29^Si MAS ssNMR spectra (Fig. S1), which indicates before and after PPA modification, Si chemical environment does not change. Combining the ^27^Al ssNMR data, we draw the conclusion that the PPA will only graft on the alumina side and leaves silica side intact, which makes surface modification totally asymmetric. We made an effort to measure the ^29^Si MAS ssNMR spectrum after ATRP reaction. However, due to the strong shielding effect caused by Cu^2+^, which was introduced during the ATRP reaction as a catalyst, no ^29^Si signal can be detected. A ^13^C MAS ssNMR spectrum was measured for the PPA-halloysite-PDMAEMA sample. The peak at 398.00 ppm is produced by the carbonyl group from PDMAEMA; the peak at 131.66 ppm is attributed to phenyl group from PPA, which again, suggests the successful surface modifications on the alumina side and silica side (Fig. S2).

**Figure 2:**
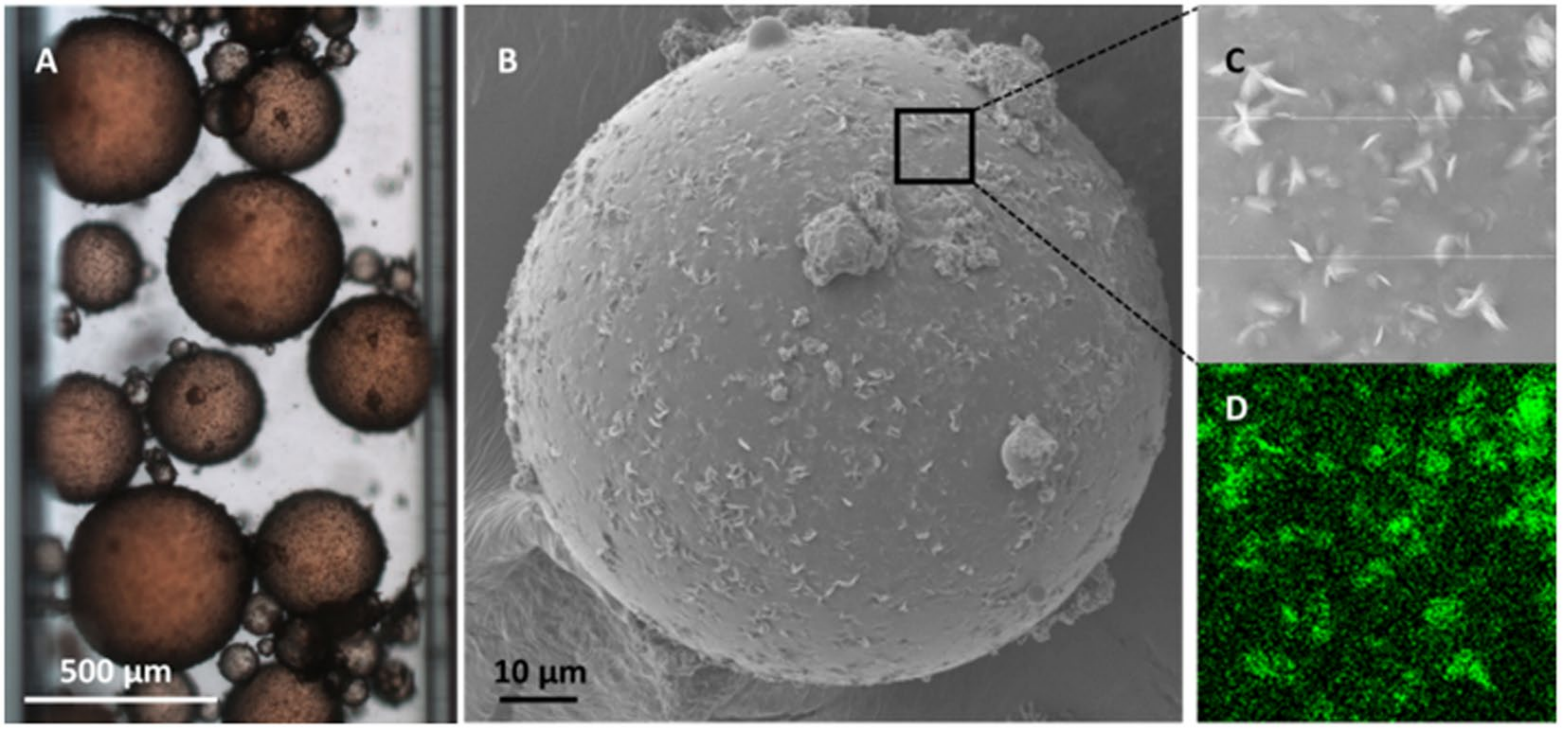
Pickering emulsion and interfaces. (**A**) Optical microscopic image of dodecane in water emulsion: the emulsion is stabilized by Janus nanoplate surfactant. Dodecane oil is dyed with 0.1 wt% Sudan IV red. Emulsion is sealed in a square capillary tube with 1-mm side length for better observation purpose. Emulsion surface is covered by Janus nanoplate surfactant. (**B**) SEM image of polymerized styrene in water emulsion: emulsion droplet is stabilized by Janus nanoplate surfactant. The droplet shows a high surface coverage of Janus nanoplate surfactant. (**C**) Magnified view of emulsion surface. (D) EDS element mapping of the magnified region, which exhibits a strong signal of Al at the interface.

The TEM and SEM images of pristine halloysite (Figs 2 and S3, respectively) present the typical morphology of the aluminosilicate clay material, which is a hollow tubular scroll with a multiple-layer wall. Because the nanoclay is processed from natural clay material directly, the diameter, length, and aspect ratio of halloysite raw material are widely distributed. The main morphology of pre-processed halloysite, however, remains as hollow nanotubes. After reacting with PPA, the hydroxyl group from the PPA forms a covalent bond with the hydroxyl group on the alumina side of halloysite and unscrolls the multiple-layer wall nanotube into a nanoplate with around 30–50 nm thickness. The morphology changes from tube to platelet due to the hindering effect caused by the inserted benzene group between layers and the weakened hydrogen bond^28,29,32,33^. After the silica-side salinization and surface ATRP reactions, the nanoplates morphology remains the same, however, some nanoplates show stronger charge issues than the PPA-halloysite under SEM, which is also an indication that a layer of polymer modification has been grafted on the surface.

### Pickering Emulsions Stabilized by Janus Nanoplate Surfactants

Dodecane/water emulsion are generated with nanoplate surfactant and captured in a capillary tube to study the performance of Janus nanoplate surfactant. Figure 2A shows typical emulsion droplets stabilized by JNP surfactant. A uniform layer of platelets assembles at the emulsion interface even at a low surfactant concentration (0.25 wt% platelet concentration was used here). These Pickering emulsions exhibit a high polydispersity mainly due to the non-uniform size of the nanoplate produced from natural clay. Despite the large polydispersity observed from the microscopic image, emulsion droplets can remain stable against Ostwald ripening during a long period of time under high temperature (Fig. 2A after 24 hours, 75 °C oven; fresh emulsion shown in Fig. S4c), which demonstrates the nanoplate surfactant’s excellent ability to stabilize emulsions. Emulsion stability is also studied under room temperature for one week (Fig. S4a,b). Comparing size distribution of fresh sample and one-week aged sample, emulsion average size dropped to 15 µm from 18 µm due to moderate coalescence (Fig. S4c).

To better observe the platelet surfactant behavior at emulsion interface, we designed a polymerizable Pickering emulsion system. Firstly, a styrene/water emulsion was prepared with platelet surfactant as the only emulsifier. Then, a styrene droplet was polymerized and observed under SEM. The SEM image of polystyrene particles stabilized with nanoplate surfactant (Fig. 2B) shows that Janus nanoplates are attached on the styrene water interface, forming a compact assembly to stabilized interface. There is no overlap between Janus nanoplates, which is consistent with spherical system^35,36^. A magnified image (Fig. 2C) at the interface shows that most nanoplates are parallel to the tangent direction of the interface, but some of the platelets are tilted. According to simulation work of other research teams^37,38^, those tilted and distorted configurations are thermodynamically unfavorable. It is possible that platelets are tilted due to polymerization shrinkage during the polymerization of styrene. In comparison, one-side modified platelet, the PPA-halloysite, fails to stabilize emulsion during polymerization (Fig. S5), although it is able to form a Pickering emulsion of styrene in water initially. The result shows that the asymmetric modification on the clay platelet is critical for it to emulsify and stabilize emulsions under high temperature. Salinity tolerance of the emulsion was also studied (Fig. S6). Results show that emulsions remain stable under 5% to 20% brine concentrations.

Energy dispersive spectroscopy (EDS) mapping is employed to further analyze platelet composition and distribution. The EDS mapping reveals the nanoplate attaching at the interface is mainly composed of Al, Si, O, and P (Figs 2D and S7), which is consistent with the aluminosilicate based, PPA surface-modified nanoplate surfactant composition. Again, this result confirms the forming of platelet assembly layer at interface. Due to low contrast, some platelets at interface cannot be observed clearly with SEM image but can be seen on the EDS mapping.

### Phase Behaviors of Oil/water Pickering Emulsion

**Figure 3:**
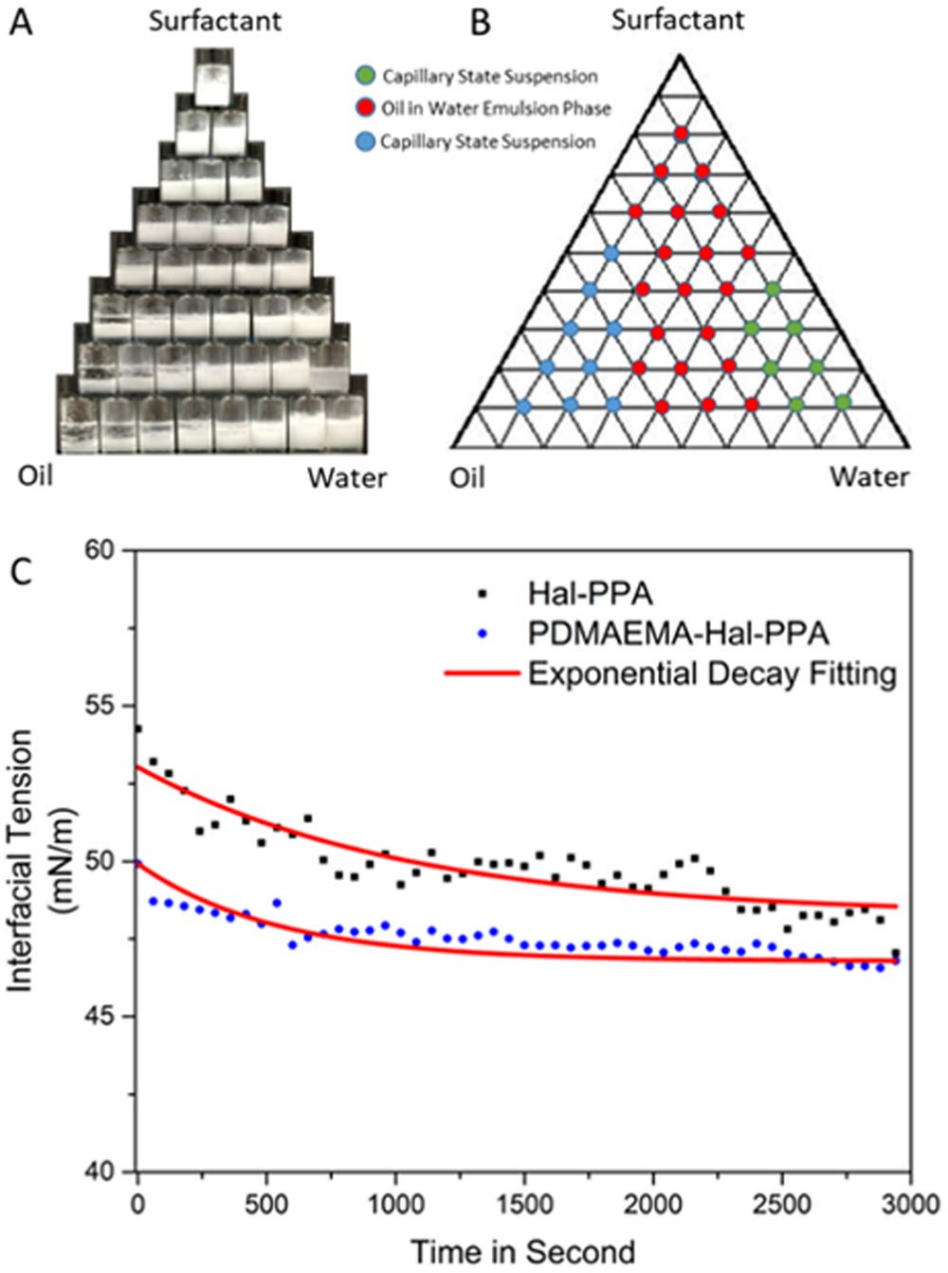
Phase behaviour and interfacial tension. (**A**) Phase behavior of dodecane/water emulsion with 0.25 wt% of Janus nanoplate surfactants. Surfactant, oil and water ratio change from 1 to 8. (**B**) Phase diagrams generated according to the equilibrium phase behavior. (**C**) Dynamic interfacial tension between dodecane and water, stabilized by Janus nanoplate surfactants and PPA-modified halloysite platelets.

Oil/water/nanoplate surfactant behaviors are studied under different compositions. Two capillary phases and an oil-in-water Pickering emulsion phase are observed in the emulsion system stabilized by the nanoplate surfactant, as shown in Fig. 3A,B. The two capillary phases correspond to oil forming a capillary phase with the hydrophobic platelet surface and water forming a capillary phase with the hydrophilic platelet surface, which is consistent with reports in the literature^39^. Dynamic interfacial tension is measured by the pendant drop method^40^. With 0.25 wt% nanoplate surfactant, interfacial tension between dodecane oil and water (Fig. 3C) is dropping from 50.0 mN/m to 47.0 mN/m (equilibrium) within the measurement. On the contrast, platelets with hydrophobic modification on only one side drop from 53.1 mN/m to 49.0 mN/m (equilibrium). In comparison, we find water/dodecane interfacial tension is reduced with the hydrophilic polymer modification on the silica side. The reduction in interfacial tension also contributes to the ultra-stability of the Pickering emulsion stabilized by nanoplate surfactants.

### Enhanced Oil Recovery with Janus Nanoplate Surfactants

**Figure 4:**
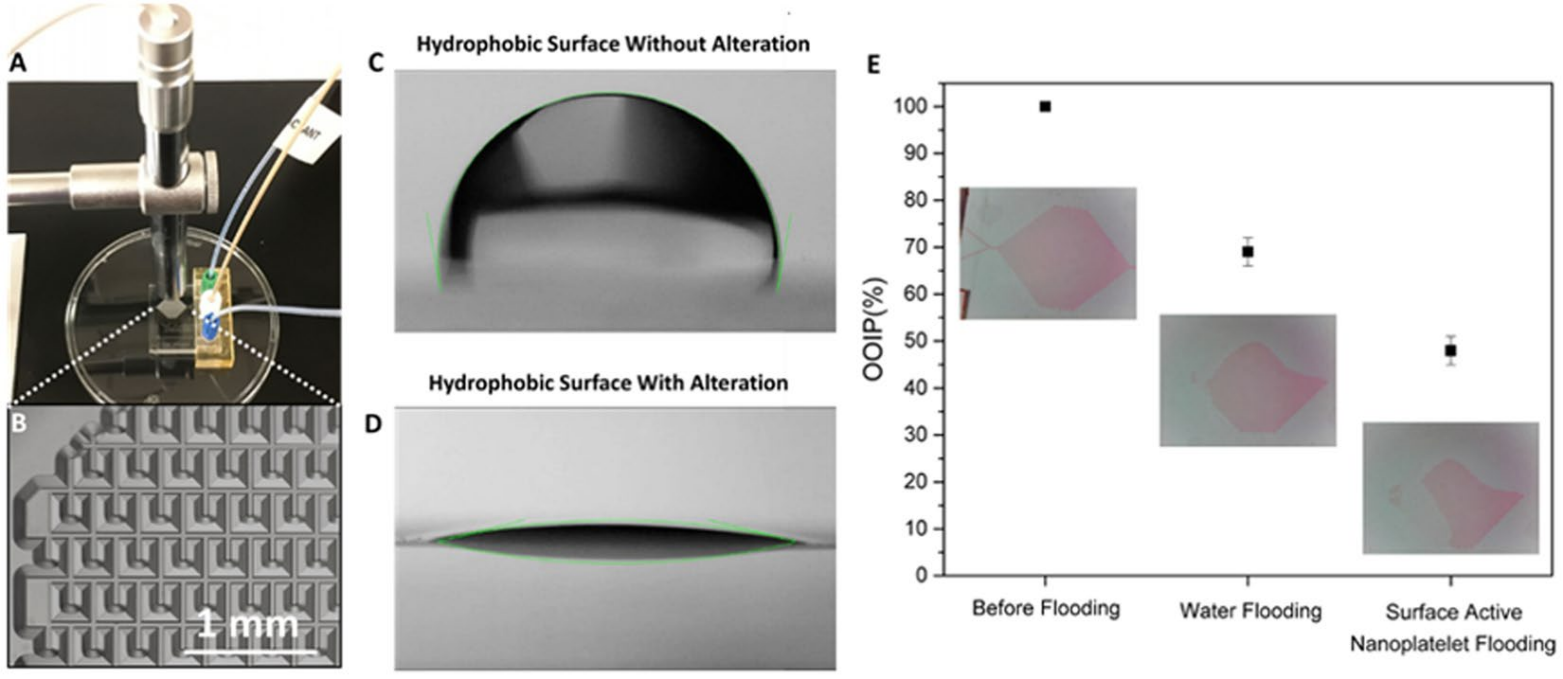
Flooding test and wettability alteration. (**A**) Water flooding and surfactant flooding test with microfluidics device. (**B**) A magnified image of microfluidics chip: the microfluidics chip has a pocket pattern with a 300 µm × 300 µm square and a 150 µm × 20 µm groove in the middle. (**C**) Contact angle measurement of hydrophobic surface before wettability alteration. (**D**) Contact angle measurement of surface after wettability alteration. (**E**) Original oil in place (OOIP) changes after water flooding followed by surfactant flooding. With water flooding OOIP reaches 69%, and with further nanoplate surfactant flooding, the OOIP percentage improves to 48%.

The chip flooding experiment was conducted with the set up shown in Fig. 4A, and a part of the chip configuration is magnified and shown in Fig. 4B. The flow rates of water and surfactant are both controlled at 0.2 ml/hour with a micro syringe pump. A maximum displacement of original oil in place, 31 ± 3%, is achieved after 1 hour of water flooding. After that, 0.25 wt% nanoplate surfactant suspension is injected into the chip again. After surfactant flooding, the total displacement of original oil in place is 52 ± 3%, which means a 21 ± 4% extra oil recovery is achieved with the Janus nanoplate surfactant (Fig. 4E).

The improved oil displacement achieved by the Janus nanoplate surfactant is attributable to the emulsifiability of the platelet surfactant and the wettability alteration property of the nanoplate. We measured changes in contact angle induced by the nanoplates (Fig. 4C,D). A glass substrate is first rendered hydrophobic with octyl triethoxysilane. The water droplet on the hydrophobic substrate contact angle is 101.0 ± 5° initially. After flooding with Janus nanoplate surfactant and dried, the contact angle is reduced to 14.0 ± 5°. Centrifugal force is proposed to be used to destabilize oil emulsion after recovery because of the specific gravity difference between oil and clay material.

## Experimental

### Materials

Halloysite nanoclay (Sigma-Aldrich™), phenyl phosphonic acid (PPA, 98%, Sigma-Aldrich™), 3-aminopropyl triethoxysilane (APTES, 99%, ACROS Organics™), triethylamine (TEA, 99%, ACROS Organics™), bromo-isobutyryl bromide (BiBB, 98%, Sigma-Aldrich™), two-dimensional Dimethylamino ethyl methacrylate (DMAEMA, 98%, Sigma-Aldrich™), ethyl α-bromoisobutyrate (ETBriB, 98%, Sigma-Aldrich™), copper monochloride (CuCl, 97%, Sigma-Aldrich™), toluene (anhydrous, 99.8%, Sigma-Aldrich™), Toluene (Certified ACS, Fisher Sci.™), dichloromethane (DCM, anhydrous, ≥99.8%, Sigma-Aldrich™), isopropanol (Certified ACS, Macron Fine Chemicals™), Sudan IV(Fisher Chemical), styrene (≥99.5%, Sigma-Aldrich™), azobisisobutyronitrile (AIBN, Sigma-Aldrich™), octyl triethoxysilane (≥97.5%, Sigma-Aldrich™) were used without further purification.

### Synthesis of Single Side Modified Halloysite

**Figure 5:**
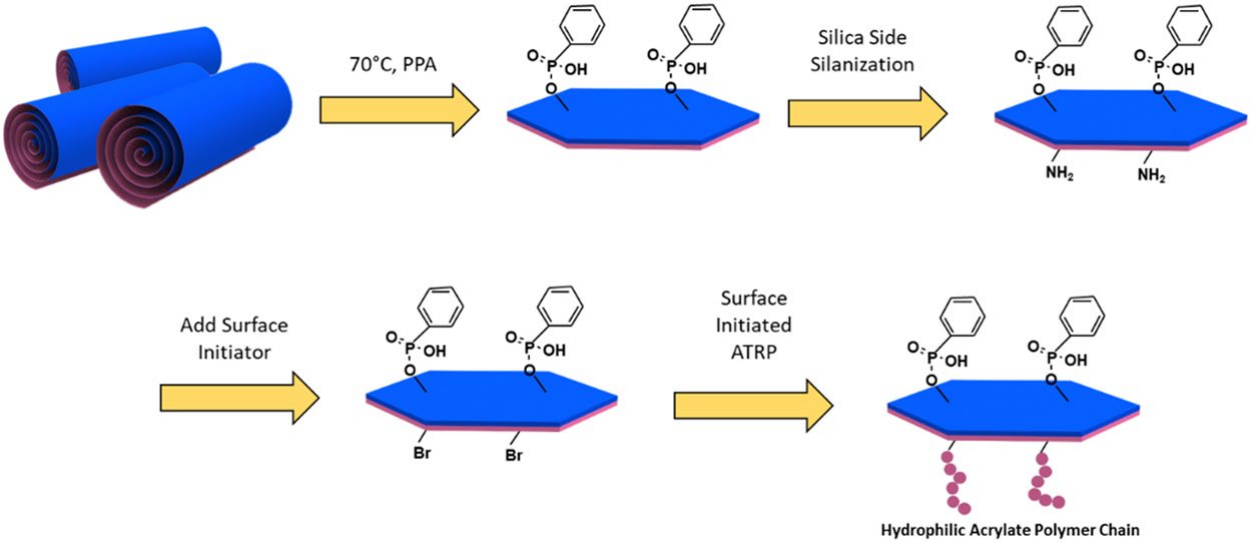
Synthesis flowchart of Janus nanoplate surfactants. Blue side represents the octahedral alumina side of halloysite and unscrolled halloysite, and magenta represents the tetrahedral silica side of halloysite and unscrolled halloysite. First, halloysite scroll is extended and grafted by PPA on the alumina side, followed with silanization on the silica side and surface-initiated ATRP reaction. The final platelet surfactant is rendered with distinct hydrophilic polymer and hydrophobic phenyl group on each side, respectively.

The synthesis procedures are summarized in Fig. 5. PPA (25 g) and halloysite (25 g) were suspended in 500 mL of deionized water (DI water) in a 1-L flask and then immersed into 70 °C oil bath with gentle stirring for 3 days. After that, the halloysite with a modified alumina side was washed three times with DI water and freeze-dried. The unscrolled halloysite with PPA modifications on one side was stocked for further reaction. The grafted PPA on the alumina side serves as a hydrophobic modification and protects the alumina side from further reactions during subsequent hydrophilic modification steps.

### Synthesis of Janus Nanoplates

The surface-initiated atomic transfer radical polymerization (ATRP) method is employed to modify the silica side of the clay platelet. To immobilize surface initiator on the silica side of halloysite clay sheet, 0.5 g PPA-modified halloysite was initially suspended in 40 ml of anhydrous toluene. Then high-power probe sonication was applied to ensure a good dispersion of nanoplates in solvent and to reduce aggregation. After sonication, APTES (10 ml) was injected into the flask with a dry syringe for salinization. The reaction was conditioned at 70 °C with constant magnetic stirring. After salinization was complete, the product was centrifuged and washed three times with toluene. For the second part of the reaction, bromide initiator grafting was conducted. In a typical procedure, 0.5 g of halloysite platelets with amino group terminals on the silica side were re-dispersed in anhydrous dichloromethane. Lastly, bromo-isobutyryl bromide (BIBB) was added to react with amino groups on the silica side. At the same time, triethylamine was added to neutralize the by-product. This step is conducted under room temperature for 3 hours. After platelets with surface initiators were prepared, ATRP was performed in isopropanol. In our surface-initiated ATRP reaction, we chose DMAEMA as monomer, EtBriB as free initiator, PMDETA as ligand, and CuCl as catalyst. The final product was washed and centrifuged with isopropanol three times, with DMF three times, and then with DI water once to remove any free polymers or monomers. The final product was freeze-dried and stocked for further characterizations.

### Materials Characterizations

The sample prepared using the above methods was characterized by transmission electron microscopy (TEM, JEOL JEM-2010) and scanning electron microscopy (SEM, JEOL JSM-7500F) under different magnifications and voltages to observe the morphology of raw halloysite precursors, unscrolled halloysite sheets, Janus nanoplates, and the polystyrene sphere surface stabilized by Janus nanoplates. Fourier-transform infrared spectroscopy (FT-IR, Thermo Nicolet 380 FT-IR spectrometer) and magic-angle spinning solid-state NMR (MAS ssNMR, Bruker Avance-400 spectrometer) were used to characterize the success of surface modifications. Interfacial tension was measured with a bench-top pendant-drop tensionmeter and OpenDrop v1.1 algorithm^40^. In a typical measurement, about 10 µL platelet surfactant suspension was dispensed into an optical cuvette, which was filled with an oil-phase solution, and image of droplet morphology was captured and processed with a computer. Contact angles were measured with a SupereEye USB microscope (Version 7.0) mounted on an optical table to demonstrate wettability alteration induced by Janus nanoplate.

### Chip Flooding Simulation

An on-chip flooding experiment was performed with a glass microfluidics model. The microfluidics model (Design 1 Solution), was manufactured with a pockets pattern design, two inlets, and one outlet. Each pocket was configured as a 300 µm × 300 µm square with a 150 µm × 20 µm groove in the middle. Sudan IV-dyed dodecane was injected as an oil phase to saturate the microfluid chip. The chip was then left aside to react with the oil for a week. DI water was then injected into the chip reservoir with a syringe pump to simulate water flooding. After the chip reached a stable condition, which means no more oil can be recovered solely by water flooding, the flooding phase was replaced with the 0.25 wt% platelet surfactant suspension. Images captured before and after platelet suspension injections were processed with ImageJ to calculate oil recovery ratio.

## Conclusions

In this work, by choosing the natural halloysite material as a model substrate, we developed a facile method to produce Janus nanoplates in bulk reaction system with a two-step modification strategy. The asymmetric modifications of Janus nanoplates on both sides were characterized in detail. Such Janus nanoplates could function as colloidal surfactants, as they demonstrate an excellent performance in emulsification and emulsion stabilization. The phase behavior of the platelet surfactant system was explored, enabling the formation of emulsion phases by changing the oil/water/surfactant ratio. Furthermore, flooding experiment with the platelet surfactant was carried out with microfluidic chips, achieving recovery of 21 ± 4% extra oil due to the emulsifiability and wettability alteration of the nanoplate surfactant. This Janus nanoplate synthesis method greatly simplifies the manufacturing process of Janus nanoplate and makes mass production of Janus particle surfactants possible. The Janus platelet surfactant exhibits an excellent emulsifiability, emulsion stability, and ability alter surface wettability, which distinguishes it from conventional molecular surfactants for high-temperature and high-salinity applications. The research disclosed here not only provides important insights for the design and synthesis of Janus colloidal surfactant, but also creates opportunities for applications in areas of biomedical, food, mining and oil industries.

## Electronic supplementary material

Supplementary Information
