# Supplementary material for: Natural Halloysites-Based Janus Platelet Surfactants for the Formation of Pickering Emulsion and Enhanced Oil Recovery
Source: Sci Rep. 2019 Jan 17;9:163. doi: 10.1038/s41598-018-36352-w (PMC6336865; doi:10.1038/s41598-018-36352-w)
Supplement: Supplementary file 1 — Supplementary Information [file 41598_2018_36352_MOESM1_ESM.docx]

**Supporting Information**

**Natural Halloysites-Based Janus Platelet Surfactants for the Formation of Pickering Emulsion and Enhanced Oil Recovery**

Lecheng Zhang,^ab^ Qun Lei,^d^ Jianhui Luo,^de^ Minxiang Zeng,^a^ Ling Wang,^a^ Dali Huang,^c^ Xuezhen Wang,^b^ Sam Mannan, ^ab^ Baoliang Peng, *^de^ Zhengdong Cheng*^abc^

^a^ Artie McFerrin Department of Chemical Engineering, Texas A&M University, College Station, TX 77843-3122, USA.

^b^ Mary Kay O'Connor Process Safety Center, Artie McFerrin Department of Chemical Engineering, Texas A&M University, College Station, TX 77843-3122, USA.

^c^ Department of Materials Science and Engineering, Texas A&M University, College Station, TX 77843-3003, USA.

^d^ Research Institute of Petroleum Exploration & Development (RIPED), PetroChina, Beijing 100083, China.

^e^ Key Laboratory of Nano Chemistry (KLNC), CNPC, Beijing 100083, China.


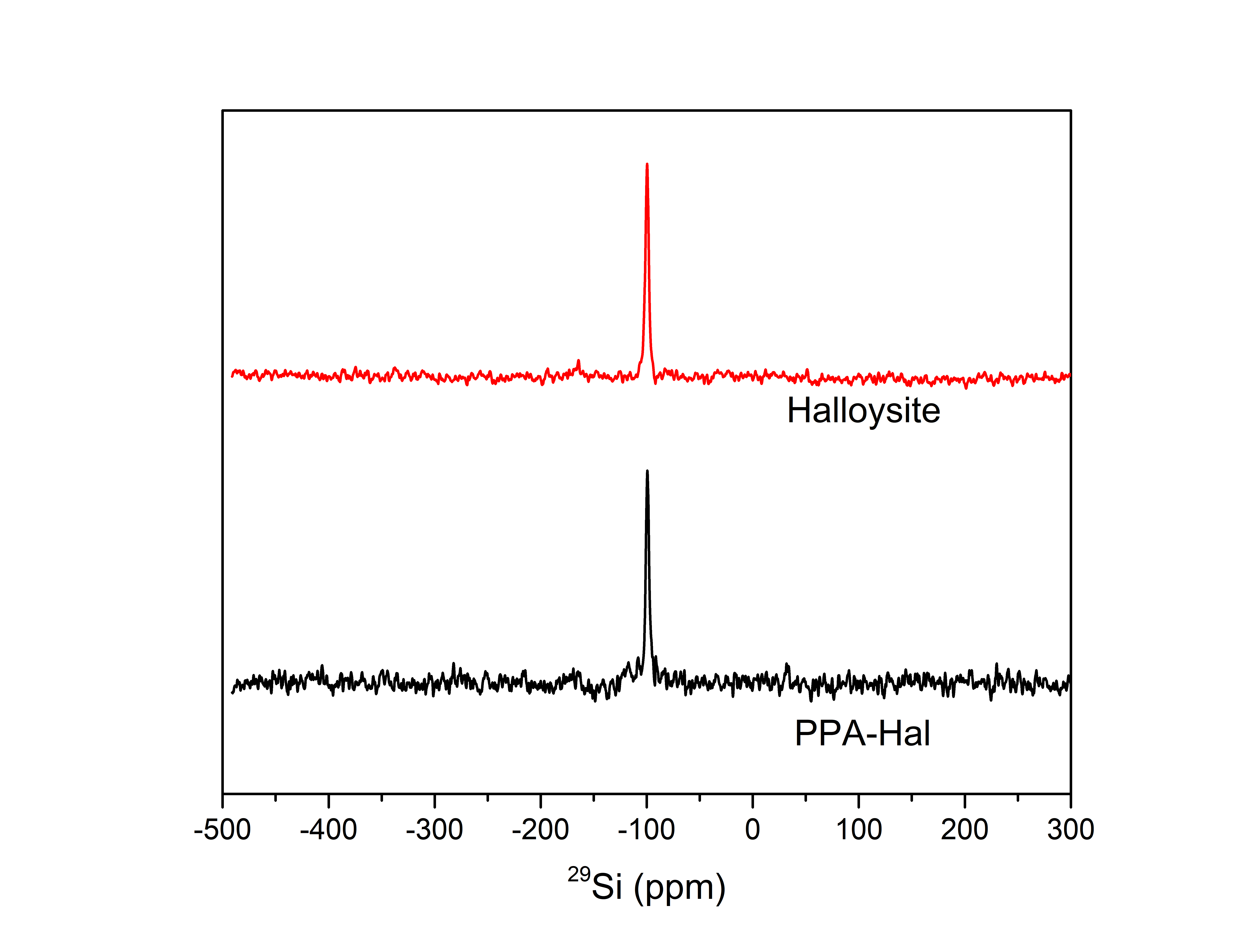


Figure S1: ^29^Si MAS NMR spectrum @ 5KHz of pristine halloysite (Top), shows a strong peak at 99.34 ppm. After alumina side PPA modification, ^29^Si MAS NMR spectrum is measured again (Bottom). No significant shift has been observed, which confirms that PPA will not react with Si during the Al side hydrophobic modification procedure.


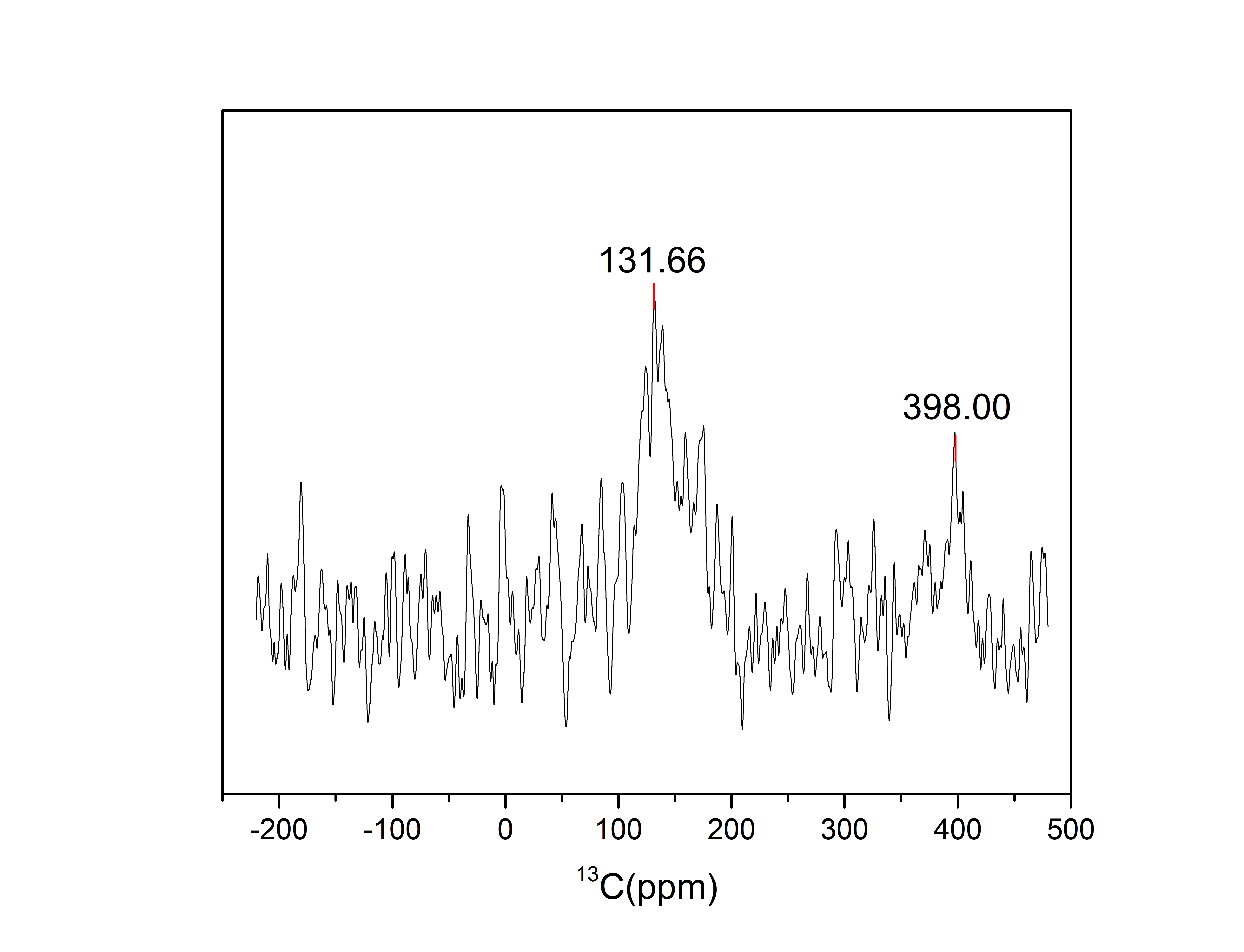


Figure S2: Solid state 13C NMR spectrum of asymmetrically modified nanoplate surfactant, it confirms the successful amphiphilic modifications on both sides of the nanoplates.


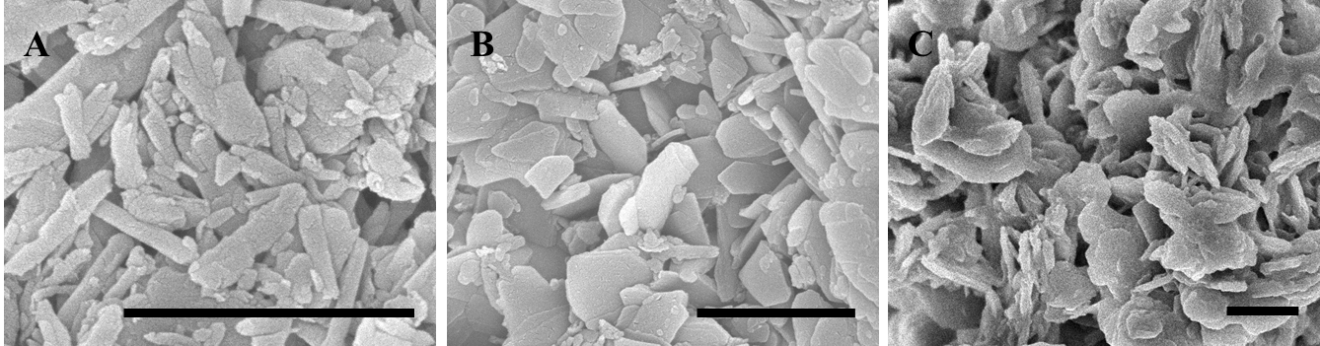


Figure S3: Scanning Electron Microscopic images. A) raw Halloysite; B) PPA unfolded Halloysite nanoplate; C) PPA-Halloysite-Poly(DMAEMA) nanoplate surfactants.


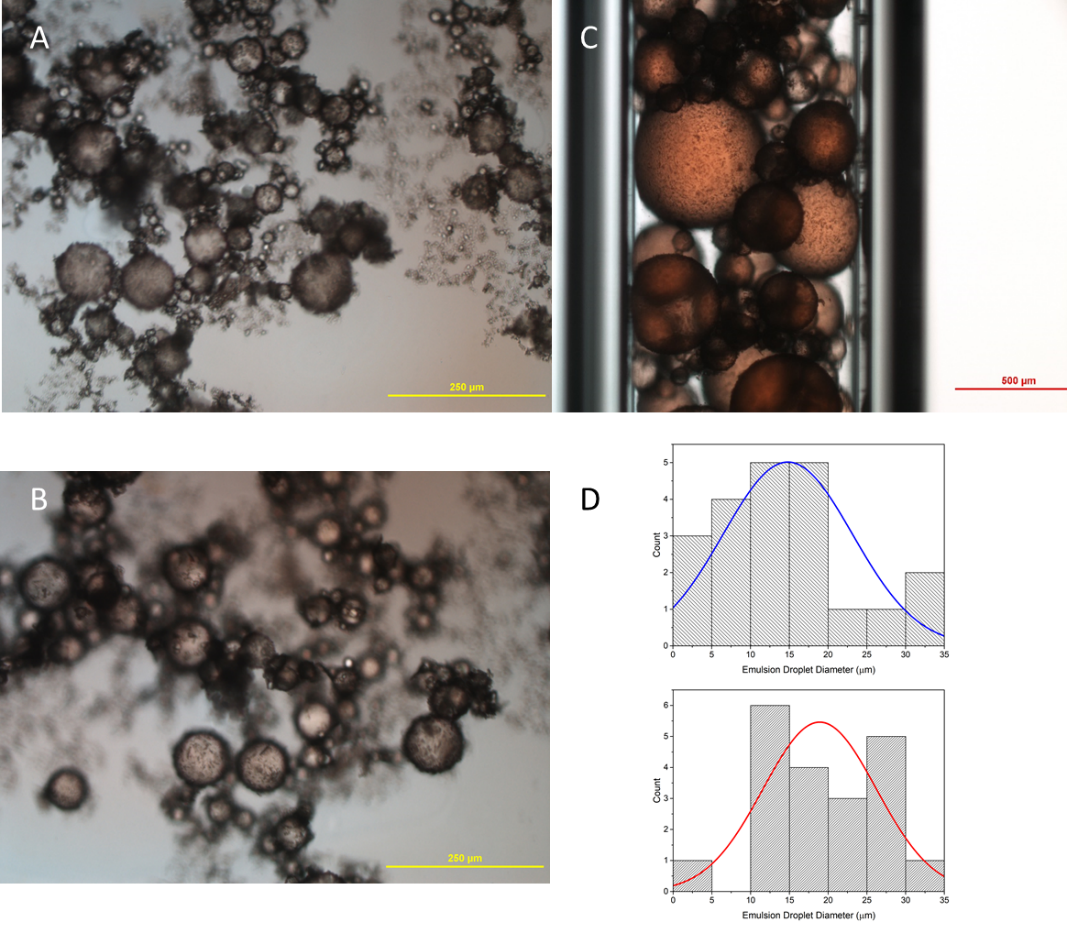


Figure S4: (a) Fresh dodecane/water emulsion stabilized by Janus nanoplate; (b) One-week aged dodecane/water emulsion stabilized by Janus nanoplate; (c) Fresh Pickering emulsion in capillary tube; (d)Size distribution of fresh made Pickering emulsion and one-week aged Pickering emulsion.


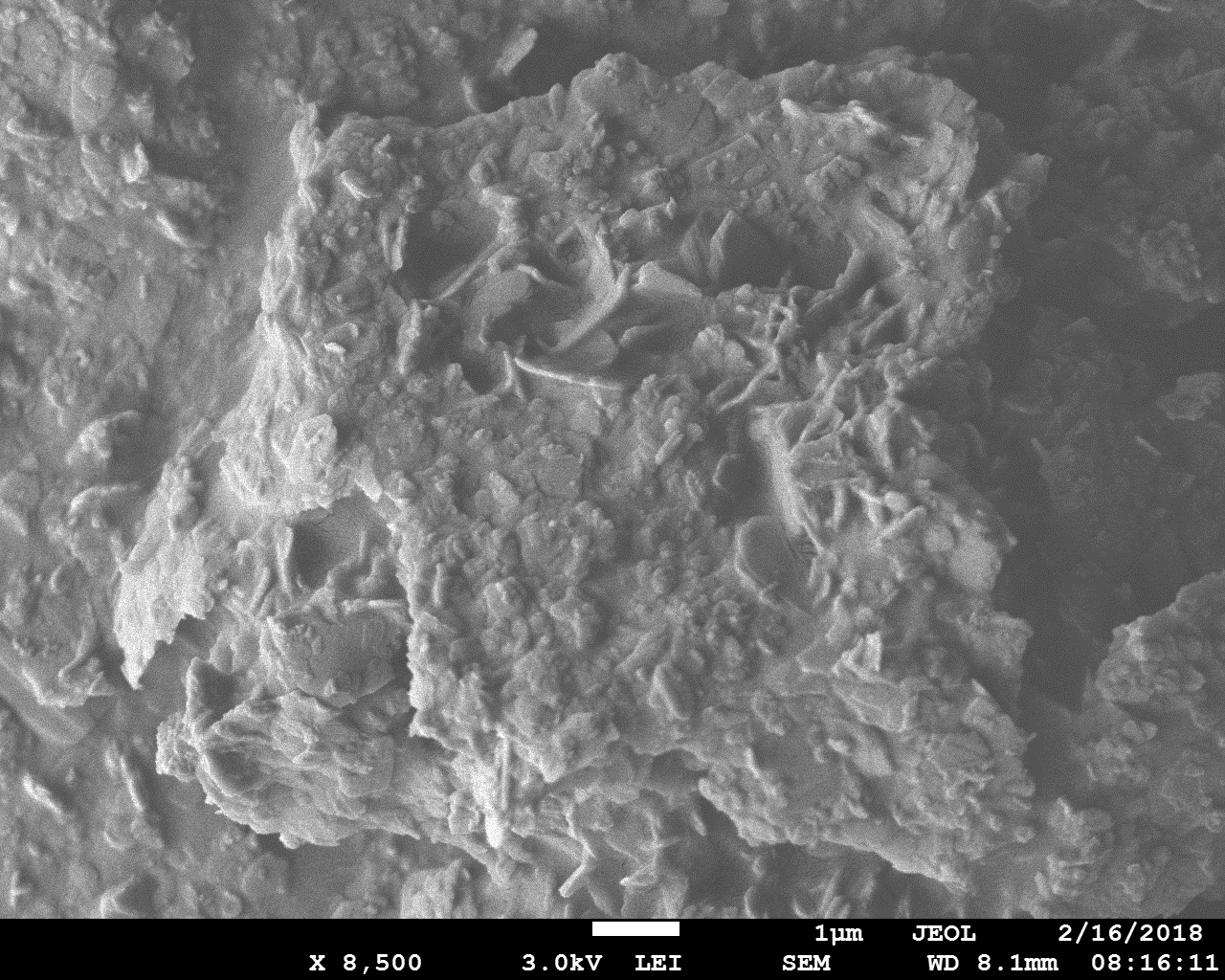


Figure S5: Styrene emulsion stabilized by one-side modified nanoplates, the PPA-Halloysite nanoplates. The spherical emulsion is destroyed during polymerization, which is carried out in a 75 °C oven for 12 hours. This comparison demonstrates the importance of double-side asymmetric modifications for emulsion stability under high temperature.


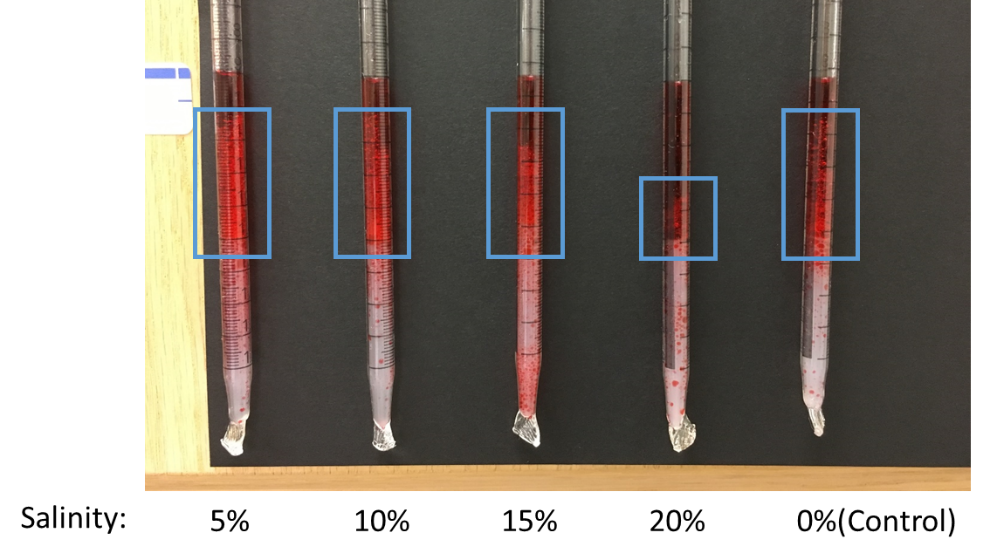


Figure S6: Nanoplate surfactant has a better performance under high salinity compared with conventional surfactants. Surfactant can still emulsify oil at 20% salinity and maintain a large quantity of emulsion at 15%.


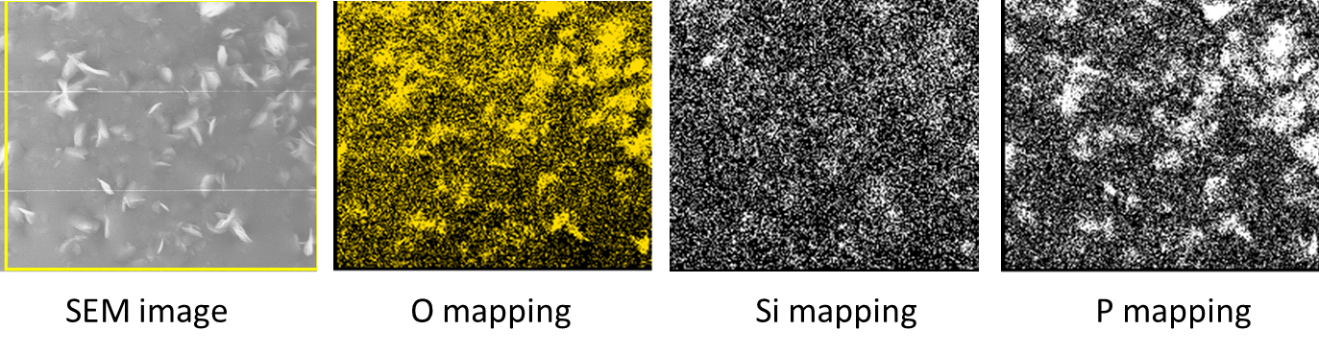


Figure S7: Energy-dispersive X-ray spectroscopy (EDS) element mapping of O, Si, P on polystyrene surface.
